# Supplementary material for: Are carbon emissions trading and green financial instruments synergistic? -Comprehensive quantitative research based on content analysis
Source: PLoS One. 2024 Mar 7;19(3):e0298601. doi: 10.1371/journal.pone.0298601 (PMC10919601; doi:10.1371/journal.pone.0298601)
Supplement: S1 Data — (ZIP) [file pone.0298601.s001.zip › Data/地级市Z_F工作报告.zip!/║┌┴·╜¡/║╫╕┌╩╨/└┤╫╘╣1⁄2╓┌║┼í╛┬φ┐╦╩2╛▌═°í┐_2010.docx]

各位代表：

　　我代表市政府向大会作政府工作报告，请审议。请政协委员和其他列席的同志提出意见。

　　一、2009年主要工作回顾

　　2009年是我市历史上极不寻常的一年，也是进入新世纪以来形势最严峻、灾害最严重、矛盾最集中、发展最困难的一年，更是我们闯关克难、共度时艰、团结奋进、逆势而上的一年。这一年，我们的城市经历了前所未有的艰难与考验。在国际金融危机严重冲击的同时，我市“集资借贷”事件集中爆发，农业旱涝灾害接踵而至，森林重大火灾连续发生，11·21特大矿难不幸降临，等等，这些事情交织、叠加在一起，给我市带来了多方面的不利影响和重大损失。面对这一切，在省委、省政府和市委的正确领导下，我们坚持“保增长、保民生、保稳定、保安全”的总体目标不动摇、不放松，勇敢面对、沉稳应对，不仅经受住了考验，顽强地走了过来，而且积累了应对多种危机的经验，增强了驾驭复杂局面的能力，取得了极其不易的成绩。

　　我市地区生产总值首次突破200亿元，完成206.3亿元，比上年增长13.9％；财政收入完成27.7亿元，增长26％，其中一般预算收入突破10亿元，完成11.7亿元，增长25.9％；固定资产投资突破百亿元，完成109.2亿元，增长51％。其中，亿元以上工业项目完成投资增幅为132.5%，列全省第一；规模以上工业增加值完成72亿元，增长20％；进出口总额完成5376万美元，增长19.8%，增幅列全省第三位；利用外资1303万美元，增长28.4%；社会消费品零售总额完成60.6亿元，增长18.9%；金融机构存贷款余额分别达到290.4亿元、207.8亿元，比年初增长32%、46%;城镇居民人均可支配收入突破万元，达到10847元，增长8.5%；农民人均纯收入突破5000元，达到5100元，按可比口径增长15%。这标志着我市上次人代会确定的主要计划指标全部超额完成，且多项指标的增幅高于全省平均水平，排位明显前移，并进入全省优秀行列，是近年来最好的一年。

　　项目建设取得重大进展，三次产业稳步发展。积极推进第二轮“三年项目建设年”，先后组织了各县区“看项目、比发展”活动、赴牡丹江“学经验、找差距、促发展”活动和项目建设“百日攻坚战”活动。全年签订招商引资项目566项，到位资金98.7亿元，增长15.8%。坚持工业立市战略，围绕优化结构上项目、促发展。重点项目加快推进，全年共实施投资千万元以上工业项目49个，比年初计划增加13个，其中投资亿元以上项目13个，正在推进和已投产的“百万级”项目达到19个。鹤翔90万吨焦化、鹤矿集团立达10万千瓦矸石热电、国电龙源公司望云峰5万千瓦风力发电、人和米业稻壳发电、宝泉岭肉业与双汇集团合建的熟食生产线等22个重点项目投产。其中，矸石、稻壳和风力发电均为我市第一个同类项目。龙煤东化百万吨化肥综合楼、化工品库等主体已完成，中化华鹤甲醇项目已完成厂区平整和煤田购置，兴汇百万吨玉米深加工二期、万源粮油深加工、双兰星药业、禾友农药搬迁改造等27个项目加快建设。区县项目竞相推进，共实施各类项目177个，其中65个投产、67个开工。园区建设全面展开，在省级工业示范基地的基础上，规划建设了市级煤电化A区、B区、医药、食品、石墨五个园区，入驻企业25户；萝北石墨科技示范园区、向阳五阳工业园区、南山工业走廊、兴安工业产业基地、东山产业基地等先后投入使用，入驻企业105户；总投资12亿元的宝泉岭经济开发区开工建设，并被省农垦总局列为“食品工业骨干区”。工业经济企稳回升。针对金融危机影响，加大对重点行业和企业的支持力度，专题召开了全市工业保增长促发展大会，出台了《鹤岗市工业保增长促发展攻坚方案》。规模以上工业企业由99户增加到114户，亏损企业由36户下降到29户，工业企业用电量11.3亿度，增长4.5%。全市生产原煤1932万吨，生产洗煤1146万吨，分别增长11%、18%，原煤入洗率达到67%。地方煤矿原煤产量348万吨，增长41%，实现税收3.1亿元，其中检顺、东兴两户企业纳税已超过或接近1亿元。烟草、啤酒、电力、白酒、通讯等企业纳税超过1千万元。建筑业恢复增长，市商品房销售面积70万平方米，增长23%；平均售价1811元/平方米，增长4.7%。坚持改革创新，围绕强化“三农”上项目、促发展。战胜了我市历史上最严重的旱涝灾害，加强科技投入和土地利用，粮食总产实现14.2亿斤，比去年增收0.9亿斤，再创历史新高。有机、绿色、无公害食品种植125万亩，增长6％，有机绿色食品标识达到35个。农业产值和增加值分别增长12%和11%，建成养殖基地50个，猪、奶牛、肉牛、羊、家禽饲养量分别增长17%、15%、15%、14%和3%。产业化经营扎实推进，龙头企业达到49户，利税增长10％，带动基地160万亩，牵动农户3.32万户，实现户均增收1000元。“米都”品牌战略加快推进，“三江平原”牌大米被铁道部指定为中国铁路系统专供大米，“实实”牌大米被省政府指定为军供大米。“鹤鸣”、“佰平”等16个商标被评为省级著名商标，特别是“梧桐”、“龙水”牌大米被国家工商总局评为“中国驰名商标”，实现了历史性突破。农村改革扎实推进，完成了27个省级试点村建设任务，组建农民专业合作组织67个，转移农村劳动力5万余人，发放农业补贴1.62亿元。新增耕地90万亩，土地流转面积达到37万亩，土地规模经营面积达到10万亩，绥滨县向日村整村耕地规模经营的经验在全省推广。启动集体林权制度改革并完成了52%的改革任务。坚持以旅游业为龙头，围绕快速壮大第三产业上项目、促发展。旅游业进入新的发展阶段。抓住我市被列入“北国风光旅游开发区”的契机，加大推动和投入力度，以“两峡一岛”为重点的景点景区建设基本完成，萝北名山镇被确定为全省重点建设的11个旅游城镇之一。接待国内外旅游者73万人次，同比增长22%，旅游业总收入2.6亿元，同比增长22%。商贸流通业取得突破性进展。一年之内，吉林温馨鸟集团名品店、河北庞大集团两个汽车“4S店”、江海证券鹤岗营业部、哈尔滨银行鹤岗分行开张营业，台湾润泰集团大润发超市、上海红星美凯龙家居广场开工建设，大连大商集团新玛特商场签约。比优特超市、宏益家电、大世界商城等本地商贸业在竞争中发展壮大。对俄合作不断加强。四次组团赴俄推动合作，锰矿、磷矿开采项目加快推进，生猪养殖、土地种植项目签订协议，林木采伐达到5万立方米。同时，我市与莫斯科华人商会等建立了友好关系，并成立了鹤岗在俄企业协会。对内合作不断拓展。与省内外50多个商会建立了联系，同湖北恩施、仙桃两州市结成友好城市，与吉林通化市签订合作协议，与丹东、吉林等市签订了《东北东部十二市（州）区域合作框架协议》。与港台合作取得突破。三江米业集团分别与台湾、香港企业签订了饲料玉米和中高端大米进入港台市场的协议，已获批6万吨出口指标；农产品市场销售网络项目在深圳注册公司。2009年，是我市园区和项目建设实效最明显的一年。

　　积极抢抓政策机遇，向上争取成效显著。在争取政策支持方面，继省委全会决定将我市列入“东部煤电化基地规划区”和“两大平原农业综合开发实验区”之后，经过不懈努力，又被纳入全省“北国风光特色旅游开发区”和“大小兴安岭生态功能区”发展规划。在争取资金支持方面，共争取到17亿元国家扩大内需资金和省配套资金支持，占全省争取资金的14%以上。其中，继龙煤东化公司获得1.6亿元支持资金后，中化华鹤公司又获得6000万元资金支持；廉租房争取资金1.1亿元，在13个地市中列第二位；土地矿山治理工程争取资金1.43亿元，在13个地市中列第一位；全市国有关闭破产企业退休人员纳入医疗保险争取资金1.2亿元。此外，还获得国家代发财政债券1亿元。在争取项目审批方面，中化华鹤公司一期化肥项目、龙煤东化公司项目股权变更等获批。特别是在国家决定三年内停止审批焦化、水泥等项目前，我市鹤翔、万昌、经纬三个百万吨焦化项目和鑫塔百万吨水泥项目抢先获批。鹤佳铁路扩能改造、鹤名铁路建设项目已纳入铁道部和省政府合作纪要，我市唯一一个出省旅客列车鹤岗至天津线开通。投资9.4亿元的富绥松花江大桥、投资9.8亿元的鹤名一级公路获批并得到省交通厅贷款担保。“鹤岗台湾农民创业园”（东北地区第一家）获得国台办和农业部批准。此外，还有涉及产业调整、社会事业和基础设施等100余个项目列入国家和省支持计划。在引进科技人才基地方面，国家重点化工矿产品实验室（全国第二家）、黑龙江省轻集料及制品质量检测研究中心、黑龙江省东部建筑节能检测中心均获筹建批复；黑龙江省石墨工程技术研究中心在我市设立；省学位办、黑科大和佳大等联建的“黑龙江省东部煤电化高级人才培训基地及黑科大研究生分院”开工建设，哈铁道学院与鹤岗师专联建的职业培训基地投入使用。第三中学被确定为全国空军招飞基地，首批向国家输送8名飞行专业学员、50名航空服务人员。此外，国家级流动人口监测站、省级体育人才后备基地等项目均获得批复和资金支持。2009年，是我市历史上争取资金和项目支持最多的一年。

　　突出抓好十项工程，百件实事扎实推进。年初确定的120件实事，计划当年完成89件，已有81件完成或超额完成，31件跨年度工程均完成计划进度。虽有8件因资金到位延迟或雨季过长等原因影响了进度，但均已开工或得到前期资金支持。在文明城市建设工程中，新建地标性雕塑“翔鹤”主题广场、10条城市道路、3万平方米街巷路、城市输变电扩容工程、消防四中队、应急处置指挥中心、第一个省级标准化社区等任务全部完成。其中义务植树355万余株，完成计划的237%，是前两年的总和。农村公路建设完成234公里，完成计划的130%。富绥松花江大桥、公共资源交易中心、人民信访接待中心、第二看守所迁建工程开工建设。绥嘉和太平沟至嘉荫河公路、居民燃气和供热设施扩建等项目均已完成工程总量的60%以上。在农业基础设施工程中，元宝山和十里河水库除险加固、东方红乡土地整理、沼气利用工程全部完成。争取农机直补资金2923万元和农机贷款4050万元，新增大型农机具1576台（套）。建成97个村屯涉及3.78万人的饮水安全工程、建成2808栋水稻育秧大棚，均超额完成任务。小鹤立河水库消险扩建和江萝灌区建设等完成年度任务。在旅游综合开发工程中，黑龙江流域博物馆、萝北界江国际大酒店、太平沟旅游接待中心正式投入使用；桶子沟植物园原始森林景区、金顶山龙掌岩石峰景区、鹤北联营红松母树林生态景区道路建设任务基本完成。月牙湖生态景区、嘟噜河湿地公园、水莲湿地公园建设前期工作完成。在生态环境保护工程中，成立了市节能减排专门机构，设立了1000万元的专项资金，化学需氧量静削减2475吨，二氧化硫静削减888吨，单位生产总值能耗下降8.6%，节能目标考核名列全省第二位。华能鹤电脱硫改造、富力矿矿井水净化利用、城市既有建筑供热计量及节能改造、五号水库水源保护区居民搬迁、4万亩黑土地水土流失治理、国家级地质矿山公园开园等任务全部完成。城市西部污水处理、垃圾无害化处理和鑫塔水泥厂搬迁项目正在建设。在教育公平发展工程中，创建6所省级标准化学校、改善3所学校校园场地、改造1000平方米危房、维修35所学校锅炉和建设第一个中小学活动中心、第一个过街天桥等任务完成。投入460万元资助贫困学生9029名，超计划118%。进一步提高教学质量，高中阶段毛入学率达到77%，高考升学率达到 85 %，职业教育就业率达到90%。在医疗卫生服务工程中，政府增投专项资金156万元，使甲型流感得到有效监控和防治。“婴幼儿健康促进计划”、计划生育家庭奖扶制度、免费为万名妇女进行健康检查、减免符合条件的残疾军人、复员军人和军烈属医疗费等政策全部落实。新建3所社区卫生服务机构，社区卫生服务人口覆盖率达到85%。3所中小学人口素质教育示范基地、每区一所惠民医疗机构全部投入使用。在文化体育繁荣工程中，电视连续剧《解放区的天》拍摄完成，20个社区图书室和40个农家书屋建成，为城乡群众免费放映785场数字电影，地面数字电视开通。市博物馆、文化活动中心开工建设。我市文艺作品获国家和省级奖项32个，电视节目在全省评比中获奖36个，均创历史最好水平。体育设施进一步完善，天水湖健身休闲广场建成，新增全民健身路径30套。竞技体育水平不断提高，获得省级以上赛事金牌79枚。其中，我市刘佳宇获得单板滑雪世界杯总冠军，李明轩获得全运会男子200米冠军，市政府出资6万元进行了表彰奖励。在人才科技强市工程中，设立60万元专项资金，对全市500多名各类人才进行了培训。适应主导产业发展需要，委托培训了178名煤化工和旅游专业人才。制发了《鹤岗市高新技术企业认定办法》，省级民营科技型企业达到59户，获得专利授权18项。组建了6个企业研发机构，三精千鹤被认定为我市首家国家高新技术企业；香港南海油集团在我市建立了石墨工程技术研究中心；万禾园油脂公司依托北京化工大学、江南大学组建了黑龙江省水稻深加工研究中心；禾友农药公司与黑龙江大学合建的农药中试基地正在申报省级基地；双兰星药业公司与省中医药大学合建的药品化验室正在申报国家级实验室。瓦斯防治等2项成果获省级科技奖，并被列为推广项目。在保障性安居工程中，沉陷区治理新建成36万平方米，近6000户；棚户区改造开工94万平方米，竣工68万平方米；农村泥草房改造3007户，完成计划的115%；廉租房开工建设3336套，已分配300套，租房补贴范围扩大到1.26万户，完成计划126%，廉租住房保障对象人均建筑面积从8平方米扩大到10平方米；一氧化碳渗出区居民搬迁安置工作启动；林区棚户区改造列入省政府规划。在直接惠民利民工程中，实施积极的就业政策，发放灵活就业社保补贴4800万元，发放公益性岗位从业人员岗位和社保补贴4173万元；扩大创业担保贷款范围，贷款额度从2万元提高到5万元，为328名创业人员发放贷款1030万元；免费对1.8万人进行了就业创业培训。全市实现新增就业再就业2.5万人，完成目标的111%，登记失业率控制在3.9%。发放养老、医疗、工伤、失业等保险金3.7亿元，增长7％；为贫困群众发放取暖补助2533万元，增加6.6%。努力提高各类补助标准，失业保险由每人每月196元提高到260元；城市低保人均补差额提高33元，达到173元，列全省第三位；农村低保人均补差额提高250元，达到900元，列全省第二位；企业退休人员养老金由898元提高到1040元，提高15.8％。启动新型农村养老保险试点，参保率达到33％，新农合参合率提高到95％；启动城镇居民医疗保险工作，参保率达到58％，列全省前列。对5000余户特困户发放物价调节基金补贴105万元，发放额列全省第五位。机关事业单位人员津补贴和社区工作者每人每月增加100元生活补贴全部兑现，成为全省第五个全部兑现津补贴的城市。此外，为老服务综合楼、妇儿活动中心、未成年流浪者保护中心、20个村8395人整村脱贫等直接惠民任务全部完成。2009年，是我市历史上在社会事业和基础设施等方面投入最多的一年，特别是在民生方面，市本级财政直接投入就达2.3亿元。省和国家级媒体对我市十项工程百件实事先后进行了100多次报道。

　　狠抓平安城市建设，安全稳定形势逐步好转。全面开展“安全生产年”活动，各类安全生产事故死亡人数下降59.2%，特别是地方煤矿死亡人数下降96%，两项排名均列全省第一，受到省政府表彰奖励。克服各种困难，按时完成了省政府下达的关闭15个矿井任务。全力支持鹤矿分公司妥善处置11·21矿难救援和善后工作；及时扑灭了多起重大森林火灾，城市消防保持了连续5年无人员伤亡记录。高度重视社会稳定，切实加大解决难点问题的力度，投入1275万元解决了83件信访积案，筹集5760万元偿还了部分历史欠账，使信访整体形势好转。进省个体访下降15%，集体访下降66.6%，进京非正常集体访为零。妥善处置“集资借贷”事件，投入了大量领导和工作力量，抽调了600多名干部，成立了94个包保组和专案组，建立了一整套比较成熟的组织、制度和政策体系，接待上访3万多人次。紧紧抓住加快还款这个主要矛盾，盘活1亿多元资金，支持企业累计还款11亿元，占全部集资额的30%（现已还款约14亿元，约占全部集资总额的40%）。加强依法处置工作，立案43起、逮捕24人、移送司法机关49人。近日，已公开对5起案件的10名被告人作出一审判决，其中1人被判处死刑，2人被判处死刑缓期两年执行，2人被判处无期徒刑。同时，高度重视解决群众实际困难，出台特困救助办法，筹资2122万元对5500名放款群众进行了大病治疗和子女就学救助，有效维护了群众利益和大局稳定。国务院处非办和省政府对我市处置工作给予了充分肯定。治安形势稳定好转，刑事案件发案率下降2.8％，破案率上升13.7%。在省公安厅的支持下，交通局长被杀案件8天告破。近日，已作出一审判决，其中2人被判处死刑，1人被判处死刑缓期两年执行，2人被判无期徒刑。近期，公安机关又打掉两个涉嫌带有黑恶性质的犯罪组织，现已逮捕37人。

　　政策措施保障有力，政府自身建设不断加强。深入开展学习实践科学发展观活动，进一步明晰了发展思路，强化了发展措施。在谋划全局和长远上，投入300多万元，编制了全市煤化工用煤、城市水源选点、城市供热、城市燃气、新能源建设、旅游产业和城乡建设等9个中长期发展规划。在强化政策措施上，完善了市级领导包项目、包企业和重点任务督查落实责任制度，制定了支持中小企业发展的13项政策、支持房地产企业发展10项措施，以及《工业经济项目考核办法》、《依法拆迁和行政拆迁办法》、《城市资源经营办法》、《城市管理实施意见》、《棚户区改造实施意见》、《促进就业工作意见》等，出台了《价格调节基金征收工作实施方案》、《新增税源企业税收缴库办法的实施意见》、《加强煤炭企业产销统计管理的实施办法》，以及《鹤岗市人民建议征集和奖励办法》、《鹤岗市市民特殊荣誉奖励办法》、《鹤岗市政府工作创新奖评选办法》等25个文件。这些组合性政策措施的及时出台，对全市经济社会发展起到了至关重要的指导和保障作用。在财政金融保障上，加大资金支持力度，通过财政担保和直接投入等形式，为企业解决贷款4.8亿元，设立了1000万元“中小企业发展专项资金”，有35户企业得到首批扶持资金740万元。加大税收力度，国、地两税税收增幅均列全省第四位。积极推进金融改革，我市首批4户小额贷款公司开业，累计为中小企业提供贷款1亿多元；成立了工商联民营经济担保商会，累计为企业提供贷款8000多万元；组建了33家农民资金互助合作社，筹集资本金2000多万元。各金融机构及时出支持地方发展措施，为全市企业和项目提供贷款154亿元，贷存比达到71%，比上年提高了7个百分点。2009年，是我市历史上获得金融机构贷款支持最多的一年。在政府自身建设上，积极推动效能建设，不断扩大政务公开，政府领导分工、部门职责和重点工作任务等全部在主要媒体和广场电子屏上公示。主动接受市人大、市政协和人民群众监督，共办理人大代表建议93件、政协委员提案213件，办复率均达98％。切实推进科学化、民主化决策进程，组建了鹤岗市“经济项目建设顾问组”和“城市规划建设专家组”，围绕经济社会发展重大问题及时召开听证会和论证会，并在媒体上专题就民生问题公开征求市民建议240多条。严格落实廉政责任制，坚持“一岗双责”，对重要决策、重要人事、重点项目安排和大额资金使用等事项，全部实行了部门审核、分管把关、会议讨论、集体决定。

　　此外，民族、宗教、妇儿、外事、侨务、审计、监察、物价、双拥、人防、司法、统计、地震、残联、老龄、档案等项事业都取得了新成绩。

　　各位代表！上述成绩的取得，是省委、省政府和市委正确领导的结果，是市人大、市政协监督支持的结果，是全市上下共同奋斗的结果。在此，我代表市政府，向全市各族人民、各民主党派、工商联、各人民团体和各界人士，向中省直各单位、驻军官兵和所有支持帮助鹤岗发展的各级领导、各方友人表示衷心的感谢和崇高的敬意！	

　　在总结成绩的同时，我们也清醒地认识到，对照科学发展观的要求，与人民群众的期盼相比，与发展较快的地区相比，我们还有较大差距。在经济发展方面，经济总量偏小，且增长过度依赖投资，消费和进出口协调拉动能力不强；发展方式相对落后，主要依靠物质特别是资源投入，科技含量、管理水平不高；产业结构层次较低，一、三产较弱，二产煤炭业独大的问题仍然突出。在财源建设方面，现有中小企业增收潜力不大，特别是鹤矿集团和地方煤矿的发展面临严峻的形势，部分新上项目尚未形成支撑能力，特别是甲醇、化肥两大项目因市场变化、产品调整及投资方变更等原因进展缓慢，短期内难以形成财力；历史欠账、各类贷款集中转化为即还债务，财政压力增大。在民生方面，城乡人民收入总体偏低，特别是低收入群体生活条件亟待改善。在安全稳定方面，多种矛盾突出，特别是“集资借贷”问题、煤矿安全问题压力巨大。在政府自身建设方面，一些部门及公职人员责任心、主动性和创造性不强，能力低、效率低，干不了事、碰不了硬、承不了重，等等。这些问题，必须着力解决。

　　二、2010年重点工作安排

　　2010年是实施“十一五”规划的最后一年，也是我市建市65周年。从总体上看，今年国家宏观政策持续稳定，经济社会发展环境会逐步好转。但是，我们必须清醒地看到，今年的形势更复杂，矛盾更突出，任务更艰巨，而且还有诸多“两难”选择的问题和不确定因素。因此，做好今年的工作，对全市上下坚定应对危机的信心，提振拼搏进取的精神，激发干事创业的热情，保持较快发展的态势，进而为实施“十二五”规划奠定良好的基础，具有重大意义。

　　按照中央、省委经济工作会议精神和市委的战略部署，今年政府工作的总体要求，可以概括为：“一调两转三快四先四保和五个更加”，即：深入贯彻科学发展观，积极落实“八大经济区”规划部署，坚持把产业结构调整作为加快发展的重要手段，把经济发展方式转变和资源型城市转型作为重大战略举措和目标，把三次产业全面加快发展作为主要任务，把“抢先建设东部煤电化能源工业基地、率先建成三江平原优质绿色食品工业基地、争先建设北部风光特色旅游胜地、优先建设高新技术产业高地”作为主导战略，把“保增长、保民生、保稳定、保安全”作为基本要求，更加注重项目建设，不断加大投资拉动力度和发展后劲；更加注重经济效益，不断增强经济增长的质量和可持续性；更加注重扩大内需，不断提高居民消费水平和消费拉动能力；更加注重协调发展，不断增进经济发展的活力和合力；更加注重改善民生，不断实现城乡居民收入持续增加和社会和谐稳定，努力实现我市经济社会更好更快发展。

　　主要预期目标是：地区生产总值和财政收入分别增长12％，固定资产投资增长35％，社会消费品零售总额、工业增加值、进出口总额、利用外资分别增长15%，城镇居民人均可支配收入、农村居民人均纯收入分别增长8％，城镇登记失业率控制在4.5%以内。

　　为实现上述目标，要重点抓好五个方面工作：

　　（一）更加突出产业项目建设，继续加大投资拉动力度，确保三年项目建设年决战决胜。将今年确定为“项目建设攻坚年”，以加快重点产业项目建设建成、投产达效为重点，在全市开展“冬季比备战、春季比开工、夏季比推进、秋季比成果”四项竞赛活动。全市争取实施千万元以上各类产业项目100项，其中亿元以上项目20项。加快煤电化基地项目建设，龙煤东化化肥项目完成铁路和水、电等基础工程，进入设备安装阶段；中化华鹤煤化工项目力争进入实质性建设。鹤翔焦化建成二期并推进下游产品项目建设，宝源焦化开工建设，万昌百万吨焦化及征楠城市燃气气源站项目力争开工；城市热电联产项目取得实质性进展，鹤矿立达矸石发电二期项目完成前期；大力支持鹤矿集团聚源煤层气项目尽快投产，鸟山煤矿复工续建，新兴、南山两矿恢复正常生产。加快食品工业基地项目建设，兴汇玉米深加工二期和万吨白酒、绥滨老窖酒业、金可达8万吨啤酒、万源米糠油深加工、万禾园油脂二期扩建、三江平原及人和米业30万吨水稻综合利用、鹤矿集团及萝北县猪牛养殖加工、家富养殖屠宰、农业部规划设计院玉米秸秆循环利用、台湾农民创业园等项目争取年内投产。加快其它重点项目建设，争取萝北石墨深加工、世达联合收割机、双兰星药业、禾友农药二期和人和、万源、泰丰三个6000千瓦稻壳发电项目投产；龙源电力新上5万千瓦风电、国能生物质秸秆发电、恒新节能灯、旭翔节能涂料、福田清洁燃料、成金煤矸石烧结砖等项目开工建设；大润发超市、红星美凯龙建材家具广场、北汽福田汽车4S店、百聚建筑装饰材料商城、萝北对俄贸易中心等项目力争年内竣工，支持哈尔滨银行、江海证券等企业做大做强。同时，抓紧谋划生成一批符合国家支持政策的新项目。坚持园区牵动战略，全面加强市、县区和大企业各园区基础设施建设，建立鹤岗园区网站，提升园区承载功能，努力实现要素集中、项目集聚、产业集群的目标。

　　（二）更加突出结构调整，着力转变发展方式，全面加快三次产业发展。通过实施资源精深加工、科技进步助推、品牌战略牵动等手段，不断促进产业结构由低层次向高水平转变，经济发展方式由粗放型向集约型转变，努力提高产业的竞争力和贡献率。一是以科学技术为先导，加快发展新型工业。围绕实施高新技术产业战略，支持企业兴建科技创新联盟和基地，切实加快技术改造和引进。着力延伸煤化、煤焦、稻米、木材、石墨、陶粒页岩等产业链，实现优势资源与高新技术强强结合；着力提升机械、造纸、医药、食品、酿酒等企业产品科技含量，实现传统产业与高新技术的升级结合；切实解决好煤、电、运、资金等制约企业生产的实际问题，着力提高经济运行质量和效益。进一步扩大财政担保中心和中小企业发展专项资金规模，加大金融机构支持和民间资本投入力度。全力支持域内大中型企业稳定发展，积极扶持焦化、矸石发电、粮食加工等新投产企业稳产达效，充分发掘啤酒、白酒、烟草、通讯、水泥、医药等骨干企业的增效潜力，努力实现热力公司、自来水公司等企业扭亏增盈，力争原煤、洗煤等企业产量稳中有升，效益不断提高。二是以农民增收为重点，加快发展现代农业。全面落实“三农政策”，积极推进“三江平原农业综合开发实验区”建设。全力抓好农业生产，围绕全省“千亿斤粮食产能工程”，种植面积稳定在325万亩以上。实施“百万亩水稻工程”，新增水稻面积33万亩，使水稻、玉米、大豆种植面积分别达到100万亩、110万亩、100万亩；有机、绿色、无公害食品种植面积达到130万亩。加快农业科技进步，新建20个农业科技园区，带动农业标准化作业200万亩，达到总面积的60%以上启动机械化整市推进工程，完成市农机大市场建设，开办农机按揭贷款和租赁业务。加快“米都”建设，发挥龙头企业和品牌作用，促进产业化、规模化发展。积极推进畜牧水产养殖业发展，抓好猪、牛及水产品基地建设，牧（渔）业增加值增长7%。积极推进城乡一体化建设，将城镇化建设与新农村建设有机结合起来，推动农村劳动力转移，放宽户籍限制，积极探索解决农民在城镇就业和落户问题。完善农业经营体制，提高集约化生产水平，全市土地流转达到40万亩，规模经营面积达到12万亩；提高农民组织化程度，新组建10个大型专业合作社。积极推进“大小兴安岭生态功能区”建设，努力争取国家和省项目支持，加快发展特色林业经济，全面完成集体林权制度改革。三是以有效扩大内需特别是居民消费为重点，加快发展现代服务业。进一步完善鼓励消费政策措施，改善消费环境，巩固传统消费，培育新的消费热点，不断提升第三产业比重。加快实施“旅游牵动”战略，积极落实“北国风光特色旅游开发区”规划，以“中俄界江游”为龙头，以“旅游开发建设服务年”活动为载体，以“鹤岗国际界江旅游节”为牵动，推动旅游产业跨越式发展。完成全市旅游发展规划编制工作，推进名山岛、细鳞河、太平沟和金顶山景区升级评定工作。全面加强宣传推介，在央视《朝闻天下》栏目等开播城市形象宣传广告，编制完成《鹤岗旅游文化大观》、《鹤岗旅游专题风光推介片》、《鹤岗旅游服务指南》、《界江之都——鹤岗邮票珍藏册》。于6月19日与中央电视台、省旅游局、俄犹太州联合举办“首届中国·黑龙江鹤岗国际界江旅游节”，组织大型文艺演出和经贸洽谈等活动，进一步提升我市旅游业的知名度和竞争力。加快实施“商业活市”战略，积极引进大型商贸物流企业，发展新型商业业态，打造惠民商务，创建商业示范社区，深入开展“万村千乡”供销连锁和“家电下乡”等活动。高标准编制鹤岗市现代物流业发展总体规划》，加快公路货运物流、食品物流、中俄农副产品物流、粮食物流、煤化工物流、煤炭物流等八个物流配送中心建设。加快实施“对外开放”战略，积极推进对俄经贸合作适应性战略调整，促进对俄经贸平稳增长。争取在俄养殖和种植项目正式启动，鹤锰公司赴俄锰铁矿开发项目实现实验性生产，境内加工项目开工建设。支持外向型出口企业巩固现有市场，开拓新市场，华翔粮油公司豆制品等进一步扩大对韩出口。加强对俄旅游合作，率先开展跨境贸易本币结算业务，建成驻俄办事中心新楼并投入使用。加强萝北口岸建设，完成集口岸通关、出口加工、边境商贸、仓储物流为一体的边境口岸经济区规划，建成中俄商品贸易大市场，推进萝北与俄十月区固冰浮桥项目。争取口岸过货增长15%，过客增长12%。四是以促进城市转型为目标，加快发展战略性新兴产业。积极推进低碳经济、循环经济、生态经济发展，制定新兴产业发展规划，大力培育新能源、新材料、新型环保、生物、信息、现代装备制造等六大战略性新兴产业。积极支持总部经济、电子商务、研发设计、文化创意、服务外包等新型服务业发展。重点抓好高档石墨制品、新型建筑材料、风电和生物质发电项目。更加注重项目和产业的税收、就业贡献和科技、环保水平，努力培育新的经济增长点，加快产业结构优化升级。

　　（三）更加突出整体优势，充分调动各方力量，促进区域经济全面协调发展。抓住国家支持老工业基地振兴，扶持革命老区、边疆地区、贫困地区加快发展和我省实施“八大经济区规划”的难得机遇，确定我市“区域经济发展”的新思路，把一切有利于加快发展的优势发挥出来，把一切有利于创造财富的积极性调动起来，努力形成竞相发展、合力发展、共同发展的新格局。进一步加快推进区域共建。以最大诚意、尽最大努力支持各大经济单元加快发展，联手打造煤电化、食品、旅游和高新技术产业基地。充分发挥鹤矿集团资源优势，重点做大做深煤炭加工业，积极发展“吃配”经济，特别是加大对本地企业用煤的支持力度，合力建好煤电化基地。充分发挥宝泉岭农垦分局资源优势和现代大农业的引领带动作用，重点深化场县合作共建，合力建好优质绿色食品工业基地。充分发挥鹤北林业局林木资源优势，与市政林业联手引进战略合作伙伴，重点加快推进在市内和俄方的深加工项目，合力建好林木产品加工基地。充分发挥华能集团行业优势，加快推进热电联产项目，合力做大煤电产业。进一步加快县域经济发展。全面贯彻全省县域经济会议精神，增强自主发展能力，加速推进农区工业化、农业产业化、农村城镇化建设，构筑以县城为龙头、中心镇为重点的新型

　　城镇体系，不断增强县域经济实力，加速构建城乡一体化格局。进一步加快城区经济发展。把城区经济发展提升到战略高度，科学制定各区发展规划，以项目建设为抓手，以壮大财力为重点，宜工则工、宜农则农、宜商则商，突出各自重点、特点和亮点，不断增强城区经济的竞争力。加快体制创新，强化区级功能，进一步放手放权，使其在经济发展、城市建设管理、公共服务和改善民生等方面发挥更多更大的作用。进一步加快非公有制经济发展。毫不动摇地支持非公有制经济更快更好发展，在全市掀起新一轮非公有制经济发展和全民创业的高潮。不断完善政策支撑体系，支持民间资本公平进入法无禁止的领域，特别是投资资源精深加工、循环经济、新兴产业、基础设施、社会公益事业等领域。开展民营企业纳税大户、支持民营经济发展有功单位和创业新星评选奖励活动，引领中小企业上规模、上水平，不断扩大非公有制经济总量。争取非公有制经济增加值增长15%、税收增长12%左右。

　　（四）更加突出改善民生，继续推进十大工程，努力实现社会和谐发展。坚持以人民群众最关心、最直接、最现实的问题为重点，努力抓好110件实事。一是继续抓好文明城市建设工程。全面掀起新一轮城市建设高潮，努力打造宜居、宜业、宜游的滨水森林城市。坚持高标准规划，完善城乡总体规划、编制城市交通组织规划及以“五山六水七区”为主的专项规划。以提高办事效率，改善发展环境为重点，进一步提高政府服务水平和社会文明程度，树立良好城市形象；以改革体制机制为动力，全面提升城市管理水平，着力解决城市卫生、供水、供热、动迁等群众反映比较强烈的突出问题，启动“净水工程”、“暖屋工程”和“环卫工程”，努力使城市的饮水更洁净、屋子更温暖、道路更畅通、空气更清新、环境更优美。积极开展创建国家“卫生城”、“旅游城”、“森林城”和“双拥模范城”等城市品牌创建活动。重点抓好27件实事。即：开工建设鹤名一级公路，建成太平沟至嘉荫河大桥公路、绥嘉兴东至太平沟段公路，完成迎宾门至南风井收费站路面升级、南山至东山区老国防路改造和南山路改造等6条公路建设任务；修建10条城市道路；完成城市西出口、北出口、东出口和北西出口路等4个出城口改造任务。完成富绥松花江公路大桥引道水稳、路面工程和兴安峻德桥、园区大鹤立河和小鹤立河桥建设任务。推进鹤佳铁路扩能改造工程和鹤名铁路前期工作。加快“五湖一河滨水工程”建设，即：完善阿陵达湖、松鹤西湖景区基础设施，启动天水湖景区改造和清源湖、鹤立湖生态景区建设工程；开工建设小鹤立河沿岸滨水景观带。同时，完成黑龙江名山段护岸工程和城区河道治理任务。加快推进“三供四化”工程建设，即：完成热力公司供热设施改扩建工程，启动饮用水提质工程，开工建设城市供水管网改造二期工程。启动鹤岗500千伏输变电工程和110千伏兴山输变电工程，开工建设110千伏园区输变电工程和110千伏峻丰输变电工程。扩建振兴广场，由1.76万平方米扩建到3.2万平方米；开工建设10万平方米大型人民广场；完善市内新世纪广场、站前广场、文化广场和儿童公园、五指山公园等基础设施。加大绿化力度，义务植树150万株，城区绿化率达到40％以上，全部完成煤矿矿区、沉陷区和麓林山植树绿化任务，乡村绿化义务植树完成5万株任务。全面更新改造市内广告牌、路街牌和交通指示标志等道路交通设施，完成13条城市道路亮化，新砌路边石6000延米、铺设人行道板5500平方米。新增7座水冲式公厕及200个垃圾箱，购置先进环卫清雪设备。建成城市规划展馆和公共资源公开交易中心。二是继续抓好农业基础设施工程。进一步强化农田水利基础设施，提高抗御自然灾害和增产增收能力，不断改善农村生产生活条件。重点抓好11件实事，即：完成萝北江萝灌区渠首和泵站工程、小鹤立河水库扩建大坝主体工程、平原和新一水库除险加固工程。完成关门嘴子水库前期工作。全部完成农村通乡、通村公路建设任务。建成50处涉及2.5万人的农村

　　饮水安全工程，自来水入户率达到70%以上。建设6处农村沼气利用工程和1400栋水稻育秧大棚。完成4万亩黑土地及水土流失治理任务。推进忠仁镇和红旗乡土地整理项目，新增耕地面积397公顷。开工建设动物疫病预防与控制中心、黑龙江流域现代农业科技示范园和马铃薯种薯繁育基地。建成绥滨李家岛网箱养鱼基地。完成24个省级示范村建设任务。三是继续抓好旅游综合开发工程。进一步完善现有旅游景区基础设施，增辟新的旅游项目，提高景点景区的可进入性和接待能力。重点抓好6件实事，即：高标准规划建设名山“旅游名镇”，完成嘟噜河湿地公园基础设施建设，完成名山高尔夫球练习场一期工程和矿史馆改造，完善太平沟、金顶山龙掌岩石峰景区、鹤北红松母树林国家森林公园基础设施建设，启动建设鹤岗市旅游集散中心，开工建设宝泉岭水莲湿地公园、月牙湖北方民族风情园、名山岛江上浴场、名山浮动码头、鹤岗市地方名优特商品专卖店和界江国际大酒店游泳馆。在哈同和鹤伊公路等地设立旅游城市宣传牌。四是继续抓好生态环境保护工程。强化节能减排目标责任制，更加注重资源与环境保护，加强对新上项目的环境评价和高能耗、高污染企业的监管，淘汰落后产能。二氧化硫排放量降低2%，化学需氧量降低12.2%，单位生产总值能耗下降6.9%，全面完成“十一五”节能减排目标。重点抓好7件实事，即：完成180万平方米既有建筑节能改造暨美容工程。建成西部污水治理及再生水利用工程，开工建设城市生活垃圾无害化处理厂，启动建设东部污水处理工程。完成兴安区沙坑矿环境治理、南山区采煤塌陷地治理、全市白灰窑污染治理、过境河流污染治理、洗煤企业污染治理和禾友农药原厂区高危污染物治理任务。继续建设岭北矿山公园，完成主体工程量的50%。建成两套大气环境质量自动监测站。争取完成鑫塔水泥新厂建设，做好搬迁准备。开工建设兴安大峡谷地质遗迹保护工程。五是继续抓好教育公平发展工程。加快推进教育改革，促进城乡、区域义务教育均衡发展，推动优质教育资源共享。全面加强师德师能建设，大力发展职业教育，提高各级各类教育综合发展水平，不断改善办学条件。重点抓好6件实事，即：翻建东方红小学、兴安四校，全部完成城市学校D级危房改造任务，启动中小学校舍抗震安全工程。建成市青少年科技活动中心、体育活动中心和2所中小学校外活动中心。开工建设市卫校实验楼和市职教中心教学楼。创建12所省级标准化学校，启动国家级重点中等职业学校创建工作。加大助学力度，资助8000名贫困学生。实施中等职业学校农村贫困家庭学生和涉农专业学生的免费政策，确保所有困难家庭特别是煤矿工人和进城务工人员子女接受正常义务教育，确保每个孩子不因贫困失学。六是继续抓好医疗卫生服务工程。推进医疗卫生体制改革，加大投入力度，以健全社区卫生、公共卫生和医疗服务体系，强化新农合医疗保障体系为主线，全面提升公共卫生服务和突发事件应急处置能力。重点抓好11件实事，即：开工建设市医疗中心暨市中医院综合大楼，加快推进三甲医院建设。启动市职业病防治院建设工程。建成市人民医院重症监护室、市妇幼保健院儿童重症监护室和中医院中药制剂室。加快推进县级医院、乡镇卫生院和村卫生室建设。改扩建2所社区卫生服务中心，社区卫生服务人口覆盖率达到90 %以上。提高农民医疗保障水平，使新型农村合作医疗人均筹资标准达到150元，参合率达到98%。建成国家级流动人口监测站和“人口与计划生育网络信息平台”，完成人口与计划生育2个县级示范中心站、4个乡级服务站和10个村服务室建设任务。全面实施“婴幼儿健康促进计划”和免费婚前医学检查政策。设立突发公共卫生事件应急储备金和计划生育基金。七是继续抓好文化体育繁荣工程。加强公共文化服务体系建设与改革，加快开发文化产业，深入实施文艺精品工程，丰富城乡群众文化生活。全面落实《全民健身条例》，加大对公共体育设施的投入力度，加快体育社会化和产业化进程。抓好第十二届全省运动会参赛工作，力争取得好成绩。重点抓好8件实事，即：完成主城区数字电视整体平移工程、电视台新闻演播室改造工程和电视高清摄录制设备升级。推进市博物馆主体工程建设，建成市地质博物馆和文化活动中心。新建10个社区图书室和48个农家书屋。加快推进乡镇文化站建设。设立文化事业发展基金。增加小区体育设施，新增全民健身路径30套，农民健身工程10项。建成国民体质监测站，创建国家级田径短跑体育后备人才基地。办好全国中学生篮球赛等赛事活动。八是继续抓好人才科技强市工程。鹤岗的发展，呼唤大批人才、渴求大批人才、急需大批人才！要切实加大投入力度，全面加快人力资源开发步伐，着力提升现有人才素质，大力培养各类实用人才，切实做好优秀人才的选拔、引进工作。充分发挥科技先导作用，不断提升自主创新能力，提高优秀科研成果落地转化率。重点抓好5件实事，即：建成“黑龙江省东部煤电化高级人才培训基地暨黑科大研究生分院”教学楼；拓宽师专与哈尔滨铁道职业技术学院联合办学渠道；完成化工矿产品、轻集料及制品和建筑节能三个检测中心办公大厦主体工程建设。开工建设市动植物异常地震宏观观测基地和水资源监控信息化工程。争取在创建国家高新技术企业、省级民营科技企业和培育国家级重点新产品方面实现新突破。九是继续抓好直接利民惠民工程。坚持把改善民生作为政府一切工作的出发点和落脚点，把就业摆上更加突出的位置，以“零就业”家庭、高校毕业生、农民工和退伍转业军人等为重点，进一步加大公共就业服务力度；以扩大范围、提高标准为重点，进一步加大社会保障力度；以贫困群体为重点，进一步加大城乡人民增加收入的力度；以“一区两棚两房”改造和建设为重点，大规模、高质量实施安居保障工程。重点抓好23件实事，即：全年新增就业实现2.2万人以上，实施“百名大学生毕业生就业创业工程”和妇女创业就业小额担保贷款财政贴息政策。将3.5万户城乡低保家庭和低收入家庭纳入医疗救助和医疗保险范畴；将1.3万名符合“家属工”和“五七工”政策条件的人员全部纳入养老保险范畴；将六区7300名国有和集体困难企业退休人员纳入城镇职工基本医疗保险范畴；将2700名“老工伤”人员全部纳入工伤保险范围。城镇居民医疗保险扩面新增3万人，覆盖率达到70％。提高企业职工失业保险标准，从 260元提高到300元；提高企业退休人员养老金标准，从1040元提高到1164元，提高11%。提高社区工作者生活补贴，现职社区主任、副主任每月提高100元并逐年增加，离岗的社区干部每月增加50元；提高城市低保补差额，从173元提高到183元，继续保持全省上游水平。逐步提高离退休干部政治和生活待遇水平。解决1万名未享受失业保险待遇的企业并轨人员失业保险金问题。为6740名60周岁农村老人发放基础养老金445万元，为城市80周岁以上低保和低收入家庭以及90周岁以上老人发放高龄老年人生活津贴。开展社区居家为老服务试点工作，服务规模达到1000人以上。完成16个村9340人整村脱贫任务。全面完成国家下达的97万平方米1.5万户沉陷区治理任务。加快棚户区改造建设，新建 82万平方米住宅小区及基础设施和配套公建工程任务。开工建设1.2万平方米林区棚户区改造工程。建设或购买廉租房1000套，廉租房租赁补贴范围扩到1.3万户。改造农村泥草房3300户，累计完成全部任务的53.7％。完成兴山、向阳地区一氧化碳渗出区居民的整体搬迁，启动南山、东山地区新渗出区治理工作。启动建设10个电气化采暖小区。建成2处省级标准化社区。增建兴安殡仪服务站。十是实施平安城市创建工程。牢固树立安全发展、和谐发展理念，真正把保一方平安做为政府的主要职责，努力提高人民群众的安全感和满意度。进一步完善体制机制，建立重大决策和重大工程社会稳定风险评估机制、维护群众权益机制，完善安全稳定责任机制，突出抓好煤矿安全、集资借贷、群众信访和社会治安工作。要进一步加强煤矿安全生产工作，严格落实责任，加大隐患整改力度，加快标准化矿井建设，坚决防止煤矿重大事故发生。积极推进现有煤矿整合，完成省政府下达的关井任务。深入开展道路交通、非煤矿山、危险化学品、消防森防、建筑施工和食品药品生产流通等重点领域专项整治，全面加强校园安全工作，坚决遏制重大安全生产和公共安全事故。要进一步加强信访工作，加大领导和投入力度，集中解决一批历史积案，切实抓好新访初访工作。要坚持依法处理“集资借贷”事件，抓住加快还款这个主要矛盾，坚决维护群众合法利益和社会稳定，进一步加大案件侦破力度，依法快侦、快诉、快审、快判一批案件。在已有支持中小企业发展12条政策的基础上，制定《支持积极还款用款企业发展的实施意见》

　　，切实加大帮扶企业发展，解决群众困难和执行还款计划的力度，力争年底前此项工作取得重大进展，让各方面满意。要进一步强化社会治安综合治理，对各种违法犯罪特别是黑恶势力坚决实行严防、严打、严惩。全面加强公安工作，努力把鹤岗公安打造成一支组织放心、群众满意、罪犯丧胆的“铁军”，坚决维护社会安定。重点抓好6件实事，即：建成人民信访接待中心。开工建设地方煤矿应急救援基地和煤矿从业人员培训基地，升级改造煤炭产量监控系统，建设地方煤矿系统安全生产局域网和安全监控调度指挥中心。加强公安基础建设和装备建设，完善城市应急指挥中心，建成公安网络监察信息平台，加强城市监控网络体系建设；实施“法眼工程”，新装400个监视器，城区重点部位覆盖率达到80%；购置大吨位高压消防车和社会抢险救援等装备，建成第二看守所。设立政法干警救助基金、信访维稳与涉法涉诉案件特困群体救助基金。

　　（五）更加突出班子和队伍建设，切实抓好机构改革，着力打造服务型政府。牢固树立“以人为本、执政为民”理念，突出加强思想、能力和作风建设。全面开展“提质提速年”活动和重点工作“倒计时推进落实责任制”。要着力提高谋划和推动科学发展的能力、应对和驾驭复杂局面的能力、发现和解决实际问题的能力、勇于和善于改革创新的能力，努力增强工作的预见性，提高工作的创造力，把握工作的主动权。要坚持把“实干”放在政府自身建设和一切工作的首位，树“实干”之理念、提“实干”之能力、用“实干”之人才、兴“实干”之风气、求“实干”之效果。要按照精简、统一、效能原则，完成全市政府机构改革任务，使政府部门从36个精简到33个。通过改革，进一步转变政府职能，强化社会管理，为加快发展创造良好的环境，为人民群众提供良好的公共服务。坚持勤俭持家、为民理财，健全财政管理体制，优化支出结构。严格控制一般性开支和公用经费，如无特殊情况，各单位一律不再追加一般性经费；严格控制会议、文件和表彰评比活动，各类会议尽量精简合并，各种评选活动原则上一个系统最多不超过一项；严格控制公费出国和公费购车，如非特殊工作需要，原则上不予安排。总之，要切实把有限的财力用在保民生、保稳定、保重点工程项

　　目上来，确保今年一百个项目、一百件实事的“双百”目标顺利完成。

　　各位代表！鹤岗正处在艰难的转型期和难得的机遇期。越是困难的时候，越要坚定信心、增强团结；越是复杂的时候，越要头脑清醒、坚韧不拔；越是落后的时候，越要变革创新、苦干实干。唯此，鹤岗才有希望，鹤岗才能发展。让我们在市委的正确领导下，以科学发展观为统领，进一步增强忧患意识、大局意识和进取意识，共同担负起重大而光荣的历史使命，集中精力，尽心竭力，多干一些惠百姓、利民生的实事，多干一些打基础、利长远的大事，为鹤岗的繁荣富强和美好明天而拼搏奋斗！
